# A Small Dose Can Cause a Major Problem

**DOI:** 10.7759/cureus.22531

**Published:** 2022-02-23

**Authors:** Pooja Patel, Adriana Yates Munar, George Michel

**Affiliations:** 1 Emergency Department, VA Medical Health Care System, Milwaukee, USA; 2 Primary Care, Waukesha Free Clinic, Waukesha, USA; 3 Rheumatology, Advocate Aurora Health, Brookfield, USA; 4 Internal Medicine, Larkin Community Hospital, South Miami, USA

**Keywords:** severe peripheral arterial disease, multivessel coronary artery disease (mvcad), side effects of medical treatment, drug overdose, overdose prevention, pain control, baclofen toxicity, baclofen, dialysis, ckd

## Abstract

Baclofen is a presynaptic gamma-aminobutyric acid agonist, which has been used to treat muscle spasms. Due to its low cost and low addiction potential, baclofen has been the muscle relaxant of choice for many years. This drug is metabolized in the kidneys, posing a threat to patients with underlying renal dysfunction, with complications ranging from drug toxicity to death. We present a case of baclofen toxicity in a patient with renal failure on hemodialysis, who presented with signs and symptoms of baclofen overdose after consuming 10 mg of the medication. The highlight of this case is the detailed history taking, verifying all home medications, and thorough physical examination of the patient.

## Introduction

Baclofen, a presynaptic gamma-aminobutyric acid (GABA) agonist, is used to treat skeletal muscle spasticity [1]. The drug was first marketed in the United States in the 1970s for the treatment of spasticity due to spinal cord disease [1]. Baclofen was a popular choice of muscle relaxant given its low cost and low tolerance potential [2]. Multiple studies over the years have demonstrated numerous off-label uses of baclofen, for example, intractable hiccups, trigeminal neuralgias, acquired nystagmus, and treatment of substance abuse including alcohol and cocaine dependency [1]. Baclofen acts via decreasing the excitatory neurotransmitter output from the spinal cord, reducing muscle tone, and hence, improving spasticity [3]. Most muscle relaxants are metabolized in the liver whereas baclofen is primarily excreted by the kidneys in its unchanged form [1]. However, the literature lacks clear dose adjustments in patients with renal dysfunction. Due to this, baclofen toxicity is found more often in patients with impaired renal function, renal failure, and/or chronic kidney disease on hemodialysis.

Over the years, multiple case reports have been published describing baclofen toxicity amongst patients with renal disorders, who are treated with higher doses of baclofen; the lowest dose of baclofen toxicity reported until now is with 25 mg of baclofen reported by Khazneh et al. [4]. We present a case of baclofen toxicity in a patient with renal failure on hemodialysis, who presented with signs and symptoms of baclofen overdose after consuming 10 mg of the medication. The highlight of this case is the detailed history taking, verifying all home medications, and thorough physical examination of the patient.

## Case presentation

A 63-year-old female patient with a complicated past medical history of end-stage renal disease on hemodialysis, complicated type 2 diabetes mellitus with peripheral neuropathy, coronary artery disease status post coronary and peripheral arterial stent placements, essential hypertension, chronic ulcers in lower extremity status post multiple wound debridement procedures, and chronic obstructive pulmonary disease was brought to the emergency department (ER) by her daughter due to new-onset confusion, drowsiness, and right lower extremity pain and infection.

On evaluation, the patient was somnolent, confused, obtunded, unable to communicate or move her extremities, and was nonverbal. The patient responded to painful stimuli. According to the patient’s daughter, the patient was recently started on baclofen 10 mg twice daily in addition to gabapentin 100 mg twice daily for chronic neuropathic pain and muscle spasms. The patient had developed an episode of confusion, drowsiness, and weakness after taking the first dose of baclofen 10 mg tablet. She was thought to be having an episode of anxiety and was administered one dose of lorazepam 2 mg intramuscularly in the ER. Following this, she was advised baclofen 5 mg prior to dialysis for the pain and muscle spasms. Due to severe pain one day prior to her current ER visit, the daughter suspected the patient must have taken baclofen 10 mg (whole tablet). Upon awakening in the morning, the patient was confused, weak, and delirious. The patient was unable to move or talk. The daughter called 911, and the patient was immediately brought to the hospital for further care and management.

**Figure 1:**
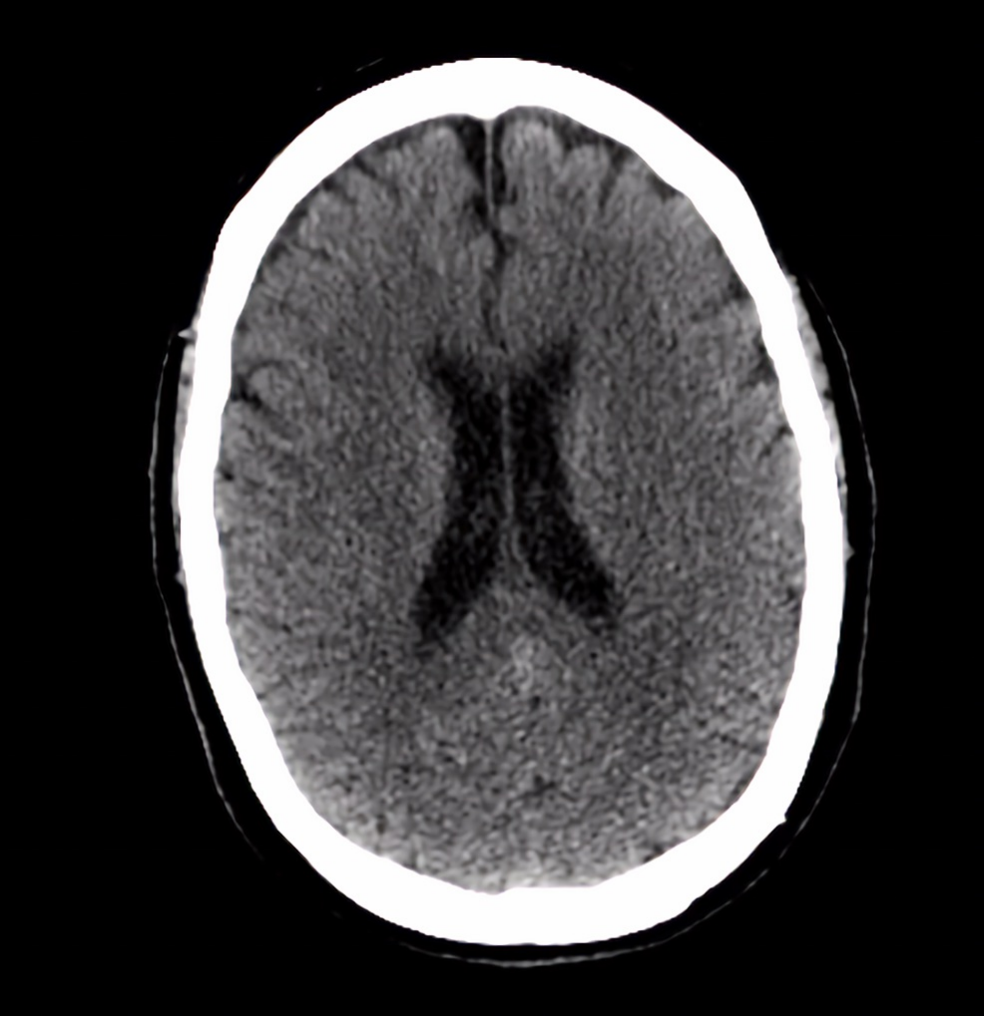
CT brain without contrast. No acute intracranial bleed, mass, or midline shift was noted.

On physical examination, her vitals were within normal limits; blood pressure of 140/88 mmHg, pulse rate of 88 beats per minute, oxygen saturation of 94% on room air, and afebrile with a temperature of 98°F. General examination revealed she was somnolent, confused, lying in bed, immobile, and in moderate discomfort. The only pertinent positive findings on her physical examination were rhonchi throughout the lung fields bilaterally with expiratory wheezing on pulmonary auscultation and a dialysis catheter in the left upper chest wall without signs of infection. Peripheral pulses were faint to palpate bilaterally in the lower extremities. She was unable to follow verbal commands or move her extremities. Hypotonia and hyporeflexia were noted on examining bilateral upper and lower extremities. The patient responded to painful stimuli. Her laboratory test results were unremarkable for any infectious or metabolic etiology for her acute encephalopathy. Chest X-ray was unremarkable for any acute cardiopulmonary changes. A CT scan of the brain without contrast was immediately ordered to rule out any intracranial pathology, which was negative for any acute changes (Figure 1).

Upon hospitalization, nephrology was consulted, and the patient underwent emergent hemodialysis. After the dialysis, the patient continued complaining of leg pain throughout the night, especially after the completion of her emergent dialysis session. She received one dose of tramadol 50 mg overnight for the pain. The following morning when she was evaluated at the bedside, she reported taking a baclofen 10 mg pill on the night prior to hospitalization due to significant and unbearable pain. The patient was educated about the risks and side effects of baclofen and her increased risk of baclofen toxicity given her history of end-stage renal disease requiring hemodialysis. She comprehended the information discussed.

## Discussion

When used at the therapeutic level, baclofen is well absorbed in the gut with oral bioavailability of 70-80% [5]. Of the absorbed baclofen, approximately 15% is metabolized by the liver via deamination [2], and the remainder 60-80% is excreted by the kidneys in its unchanged form [6]. Baclofen is rapidly absorbed in therapeutic doses to reach the maximum blood level in one to two hours, with a first-order elimination half-life of three to 6.8 hours in a patient with normal renal function [6]. In patients with chronic kidney disease (CKD), however, the half-life elimination decreased to 14.1 hours, especially in severe CKD patients [7]. Predominantly, passive glomerular filtration helps with the renal clearance of the drug [1].

Some of the common symptoms of baclofen toxicity that have been reported in the literature and various case reports are encephalopathy, seizures, coma, areflexia, muscle hypotonia, hyporeflexia, hypothermia, and respiratory and cardiovascular depression, with occasional clinical symptoms mimicking acute cerebrovascular accident or brain death [1]. There have been reports of patients developing delirium, coma, and/or seizures with the use of more than 200 mg of baclofen, requiring long-term hospitalizations and ICU admissions [8].

In patients with normal renal function, the standard dose of oral baclofen is 5 mg three times a day, which can slowly be titrated up by 15 mg a day every three to five days, to a maximum of 80 mg daily in divided doses [1]. Though the manufacturer's label does mention the cautious use of baclofen in patients with the renal disorder, there is no specific dose adjustment described based on the renal function. Some reports mention avoiding baclofen in patients undergoing hemodialysis as even the initial low doses of the drug for a short duration have led to toxicity [8]. Despite the limited data available, experts recommend avoiding baclofen altogether in all patients with estimated glomerular filtration rates (eGFRs) less than 30 mL/min/1.73 m^2^ [1,9].

Due to the small molecular weight, low volume of distribution, and relatively low protein-binding capacity, baclofen is suitable for clearance via dialysis [1]. Overall, the management of patients with baclofen toxicity is supportive, consisting of intravenous fluids administration, enteral charcoal use, and respiratory support.

Our patient had consumed 10 mg of baclofen resulting in encephalopathy, hyporeflexia, muscle hypotonia, and somnolence. After obtaining a collateral history from the patient’s family member, the source of her symptoms was identified. Immediate hemodialysis was provided, alleviating her symptoms. The patient was educated regarding the risks and side effects of continued use of baclofen and recommended considering alternate therapy for her muscle spasms.

## Conclusions

Baclofen is a commonly prescribed medication by primary care providers and specialists, especially for muscle spasms. Clinicians should reassess the patient's past medical history, allergy history, and list of home medications during each office visit as well as during hospitalization. Based on the risk versus benefit, a decision should be made whether the drug should be continued or not. If a patient is taking a medication that can cause acute withdrawal symptoms or puts the patient at risk of acute toxicity, especially in a patient with end-stage renal disease, they should be closely monitored. Patient education, including the benefits, risks, and side effects of all the prescribed medication, is very important in providing optimal patient care.
